# Transvaginal repair of Neobladder Vaginal Fistula with Martius Flap

**DOI:** 10.1590/S1677-5538.IBJU.2018.0154

**Published:** 2020-07-31

**Authors:** Daniela Carlos, Nitya Abraham, Tian C. Zhou, Michael Hung

**Affiliations:** 1 Albert Einstein College of Medicine Montefiore Medical Center NY USA Montefiore Medical Center/Albert Einstein College of Medicine, NY, USA

## Abstract

**Introduction:**

Neobladder vaginal fistula (NVF) is a known complication after cystectomy and orthotopic diversion in women, occurring in 3-5% of women. Possible risk factors for fistula formation include compromised tissue vascularity due to surgical dissection and/or radiotherapy, suture line proximity, local tissue recurrence, and injury to the vaginal wall during dissection. The surgical repair of a NVF can be challenging secondary to vaginal shortening, atrophy, local inflammation from chronic exposure to urinary leakage, and the proximity of the neobladder to the anterior vaginal wall. In this video, we present transvaginal repair of a NVF with Martius flap interposition.

**Materials and Methods:**

This is the case of a 47 year old woman with a history of radical cystectomy and creation of a Studer pouch secondary to bladder cancer two years prior who subsequently developed a NVF. Evaluation included an office cystoscopy which demonstrated a 3-4mm left-sided neobladder vaginal fistula at the level of the ileal-urethral anastomosis. No pelvic organ prolapse or evidence of bladder cancer recurrence was appreciated.

**Results:**

A vaginal approach for the NVF repair was performed with a Martius flap interposition. A water-tight closure was achieved without any intraoperative or immediate postoperative complications. The urethral Foley was removed at 2 weeks and by 4 weeks the patient did not report any urinary leakage.

**Conclusions:**

Neobladder vaginal fistula is a rare complication following cystectomy and orthotopic urinary diversion that can be repaired using a transvaginal approach. A Martius flap interposition is important to augment success of the repair. If a transvaginal approach fails a transabdominal approach or conversion to cutaneous diversion may be necessary.

## INTRODUCTION

Radical cystectomy is recommended for localized or recurrent non-invasive bladder cancer in patients who are good surgical candidates. Subsequent urinary diversion consists of an ileal conduit or various reservoirs including an orthotopic neobladder that mimics the physiologic process of micturition (1, 2). The Studer orthotopic neobladder is created using a 60- to 65cm segment of terminal ileum reconstructed into a U-shaped reservoir which is connected proximally to the ureters and distally to the urethra. This type of diversion offers good functional outcomes without compromising oncologic control.

Neobladder vaginal fistula (NVF) is a known complication after cystectomy and orthotopic diversion in women, occurring in 3-5% of women (3, 4). Possible risk factors for fistula formation include compromised tissue vascularity due to surgical dissection and/or radiotherapy, suture line proximity, local tissue recurrence, and injury to the vaginal wall during dissection. The surgical repair of a NVF can be challenging secondary to vaginal shortening, atrophy, local inflammation from chronic exposure to urinary leakage, and the proximity of the neobladder to the anterior vaginal wall. In this video, we present transvaginal repair of a neobladder vaginal fistula with Martius flap interposition.

## MATERIALS AND METHODS

This is the case of a 47 year old woman with a history of bladder cancer status post radical cystectomy, and creation of a Studer pouch two years prior who subsequently developed a neobladder vaginal fistula. She developed postoperative urinary incontinence within the first year requiring continuous pad usage with subsequent poor quality of life. She underwent a CT scan that did not demonstrate any pelvic recurrence or lymphadenopathy as well as an exam under anesthesia, cystoscopy, and pouchogram. Cystoscopy demonstrated a 3-4mm left-sided neobladder vaginal fistula at the level of the ileal-urethral anastomosis. No pelvic organ prolapse or evidence of bladder cancer recurrence was appreciated.

The procedure was begun by first performing a cystoscopy, identifying the fistulous tract, and placing a 10F Foley in order to be able to identify it throughout the case. The anterior vaginal wall was then infiltrated with lidocaine with epinephrine and an inverted U-shaped incision was made in the vaginal epithelium with the fistulous tract at the apex of the incision. The underlying vaginal epithelium was dissected off the muscularis circumferentially around the fistula. Once there was adequate mobilization around the fistula circumferentially, the fistula was closed with interrupted 4-0 PDS suture on RB-1 needle. After the bladder was closed, the muscularis was mobilized by sharp dissection in order to cover the incision while avoiding overlapping suture lines. A watertight closure was appreciated by distending the bladder with diluted methylene blue. A left Martius flap was harvested by making a vertical incision on the left labia majora and mobilizing the subcutaneous fat pad. The superior pedicle was divided leaving the inferior pedicle with adequate blood supply. The pedicle was then tunneled subcutaneously towards the vaginal incision and interposed over the vaginal muscularis to provide another layer of closure using interrupted 4-0 Vicryl® stitches. The vaginal epithelium was closed in a running-locked fashion with Vicryl® suture. Hemostasis was achieved at both vaginal and groin incisions. The groin incision was closed in a subcuticular fashion with 4-0 Monocryl. A Penrose drain was left within the subcutaneous space at the groin site. The following day the vaginal packing and Penrose were removed when drainage was no longer occurring.

## RESULT

A vaginal approach for the NVF repair was performed with a Martius flap interposition. A water-tight, three layered closure was achieved without any intraoperative or immediate postoperative complications. After two weeks, the urethral Foley was removed and the patient reported no leakage from vagina. She developed retention requiring catheterization and only reported incontinence at night. At 4 weeks post-op the nighttime incontinence resolved after evening fluid restriction. She had no daytime incontinence. At 6 months follow-up the patient reported stress urinary incontinence 2-3 hours after catheterizing the bladder. She was offered urethral bulking agent injection but deferred at this time.

## DISCUSSION

The risk of developing a NVF is estimated to be 3-5% of women who have undergone cystectomy and orthotopic diversion. In addition, after the creation of a neobladder, high rates of some form of voiding or storage dysfunction occur (5–8). Based on several functional outcome studies, 56-58. 0% of the patients require clean intermittent self-catheterization to facilitate bladder emptying. Stein et al. (9), reported frequent leakage or no urinary control whatsoever during daytime in 23% of women as well as nocturnal urinary incontinence reported by 34%. Even though functional complications appear to be common, compared to ileal conduits, women who have undergone neobladder diversion report improved quality of life due to better self-confidence as well as restoration of leisure and professional activities (8, 9). Since urinary incontinence can be an expected finding after a neobladder creation, a neobladder vaginal fistula must be ruled out in this clinical setting.

Given the distal location of the fistula at the level of the bladder neck and risk of abdominal adhesions from her neobladder surgery, we preferred a vaginal approach. In addition, she had no prior pelvic radiation and this was the first attempt at repairing the fistula, further supporting this approach. If the vagina is not capacious, an episiotomy can be made to improve access to the fistula.

## CONCLUSION

Neobladder vaginal fistula is a known complication following cystectomy and orthotopic urinary diversion that can be repaired using a transvaginal approach. A Martius flap interposition is important to augment success of the repair. If a transvaginal approach fails, a transabdominal approach or conversion to cutaneous diversion may be necessary (10).

### Consent

Written informed consent was obtained from the patient for collection of data and publication of this Video article.

## ABBREVIATIONS

NVF: neobladder vaginal fistula
BCG: Bacillus Calmette-Guérin
